# Autonomy as Conveyed by Food Environments: Nourishment vs Restriction Among Neurodiverse Older Adults

**DOI:** 10.1093/geroni/igaf122.2325

**Published:** 2025-12-31

**Authors:** Sophie Koestner, Shari Brotman, Laura Pacheco, Aglaé Mastrostefano, Marie-Hélène Deshaies, Camille Demers, Daniel Dickson, Samuel Ragot

**Affiliations:** University of California, Los Angeles, Los Angeles, California, United States; McGill University, Montreal, Quebec, Canada; Memorial University of Newfoundland, St. John’s, Newfoundland and Labrador, Canada; McGill University, Montreal, Quebec, Canada; Université Laval, Quebec, Quebec, Canada; Université du Québec à Montréal, Montreal, Quebec, Canada; University of Saskatchewan, Saskatoon, Saskatchewan, Canada; McGill University, Montreal, Quebec, Canada

## Abstract

Food serves as a lens to understand identity, memory, and belonging. However, in the context of care, food often reflects a mechanism of social control (Davies, 2007; Gerber, 2020). Feminist scholars have critiqued conceptualizations of autonomy as “self-sufficient independence” for overlooking how individuals and their contexts are intertwined (Salami & Lashewicz, 2015, p. 93). Narrow conceptualizations of autonomy, which emphasize functional independence, hold sway in many caregiving environments, shaping food experiences (Dickson et al., 2022; Boyle, 2008). This study explores food-related experiences among neurodiverse older adults (NDOA) in Quebec. We collected intersectional life story narratives (Brotman et al., 2021) of 21 neurodiverse older adults, 15 family caregivers, and 27 service providers about challenges encountered and agency enacted across the life course. Findings highlight the interplay between NDOAs’ food experiences, identities, memories, and relationships. Our observations reveal how formal and informal carers across sectors enact controls over food consumption, schedules, and daily choices, restricting NDOAs’ autonomy. We illuminate structures of dominance and expressions of resistance, highlighting how NDOAs internalize messages conveyed through their food experiences, leading to feelings of empowerment or constraint. Findings highlight how experiences of nourishment versus restriction affect neurodiverse people into older age. We encourage carers across settings to support autonomous eating to promote agency and well-being. Such inclusive recommendations would serve to safeguard NDOAs’ autonomy and rights.

